# Racial disparities in the use of blood transfusion in major surgery

**DOI:** 10.1186/1472-6963-14-121

**Published:** 2014-03-11

**Authors:** Feng Qian, Michael P Eaton, Stewart J Lustik, Samuel F Hohmann, Carol B Diachun, Robert Pasternak, Richard N Wissler, Laurent G Glance

**Affiliations:** 1 Department of Health Policy, Management, and Behavior, University at Albany School of Public Health, One University Place, GEC 169, 12144-3445 Rensselaer, NY, USA; 2 Department of Anesthesiology, University of Rochester School of Medicine, Rochester, NY, USA; 3 Principal Consultant, Comparative Data & Information Research, University HealthSystem Consortium, Chicago, IL, USA; 4 Department of Health Systems Management, Rush University, Chicago, IL, USA

## Abstract

**Background:**

Racial disparities in healthcare in the United States are widespread and have been well documented. However, it is unknown whether racial disparities exist in the use of blood transfusion for patients undergoing major surgery.

**Methods:**

We used the University HealthSystem Consortium database (2009-2011) to examine racial disparities in perioperative red blood cells (RBCs) transfusion in patients undergoing coronary artery bypass surgery (CABG), total hip replacement (THR), and colectomy. We estimated multivariable logistic regressions to examine whether black patients are more likely than white patients to receive perioperative RBC transfusion, and to investigate potential sources of racial disparities.

**Results:**

After adjusting for patient-level factors, black patients were more likely to receive RBC transfusions for CABG (AOR = 1.41, 95% CI: [1.13, 1.76], p = 0.002) and THR (AOR = 1.39, 95% CI: [1.20, 1.62], p < 0.001), but not for colectomy (AOR = 1.08, 95% CI: [0.90, 1.30], p = 0.40). Black-white disparities in blood transfusion persisted after controlling for patient insurance and hospital effects (CABG: AOR = 1.42, 95% CI: [1.30, 1.56], p < 0.001; THR: AOR = 1.43, 95% CI: [1.29, 1.58], p < 0.001).

**Conclusions:**

We detected racial disparities in the use of blood transfusion for CABG and THR (black patients tended to receive more transfusions compared with whites), but not for colectomy. Reporting racial disparities in contemporary transfusion practices may help reduce potentially unnecessary blood transfusions in minority patients.

## Background

Racial disparities in health and health care have been well documented in the literature. Reducing racial disparities has been consistently highlighted as a national priority. Black Americans have a 50% higher age-adjusted mortality rate than white Americans [1]. Differences in health care between races are a major contributor to this striking difference in mortality outcomes [2,3]. Compared with whites, blacks are less likely to receive invasive cardiac interventions [4,5], high-cost surgical procedures [6,7], effective preventive care [8,9], medically necessary mental health services [10,11], and new medical technologies [12,13]. By contrast, black patients have significantly higher rates of interventions suggestive of less than optimal management of chronic diseases such as bilateral orchiectomies and lower-extremity amputations [14,15]. Numerous studies have documented that blacks are more likely to obtain care from lower-quality physicians and lower-quality hospitals [3,16-23]. Despite national efforts to reduce racial disparities over the last two decades, the AHRQ-funded National Healthcare Disparities Report calls attention to the fact that racial gaps in health care access and quality are not improving [15]. Given that racial and ethnic minorities currently constitute about a third of the U.S. population and are expected to make up more than half the population in 2050 [24], improving overall health outcomes cannot be accomplished without substantially improving outcomes among non-White patients [25,26].

Recent studies have focused attention on the wide variability in transfusion practices in surgery, and the association between blood transfusion and adverse outcomes. In 2007, over 15 million units of allogeneic red blood cells (RBC) were transfused in the United States with the majority administered to patients undergoing surgery [27]. Once considered a relatively safe procedure, blood transfusion is associated with increased risk of mortality and major complications in surgical patients [28-38]. However, it remains unclear whether there are differences in the use of blood transfusion between black and white patients undergoing surgery.

To our knowledge, no study has examined whether minority patients undergoing major surgery are more likely to receive blood transfusion. The primary goal of this study was to determine whether black patients undergoing major surgery were more likely to receive perioperative RBC transfusion compared with white patients. We used national all-payers data from major academic medical centers in patients undergoing three common surgical procedures with significant transfusion rates: coronary artery bypass surgery (CABG), total hip replacement (THR), and colectomy. If black-white differences in blood transfusion were found to exist, our secondary goal was to determine whether the basis for black-white differences in blood transfusion was due [1] to higher rates of blood use in black-serving hospitals, or [2] to higher rates of blood use in black patients compared to white patients treated at the same hospital.

## Methods

### Data source

The source of data was the University HealthSystem Consortium (UHC) database (January 2009 - September 2011). The time frame was chosen to represent the most contemporary data available and to minimize the effect of transfusion practice change over time. The UHC is a national alliance of academic medical centers and their affiliated hospitals. The UHC database consists of administrative records for all hospitalizations in its member institutions. This represents about 90% of the nation’s hospitalizations at academic medical centers. The UHC database includes patient demographic characteristics, admission status (emergent, urgent, elective, and trauma), ICD-9-CM diagnostic codes, ICD-9-CM procedure codes, present-on-admission (POA) codes, 3 M APR-DRG severity of illness, AHRQ comorbidities [39], in-hospital death, cost, and detailed blood use information. The race information in the UHC database is comprehensive and complete. A unique strength of the UHC database is that it contains full inpatient transfusion information [40]. The UHC dataset also includes encrypted hospital identifiers. No specific patient, physician, or hospital was examined in the analysis, and the study proposal was granted exemption by the University of Rochester Research Subjects Review Board.

### Study population

**Figure 1:**
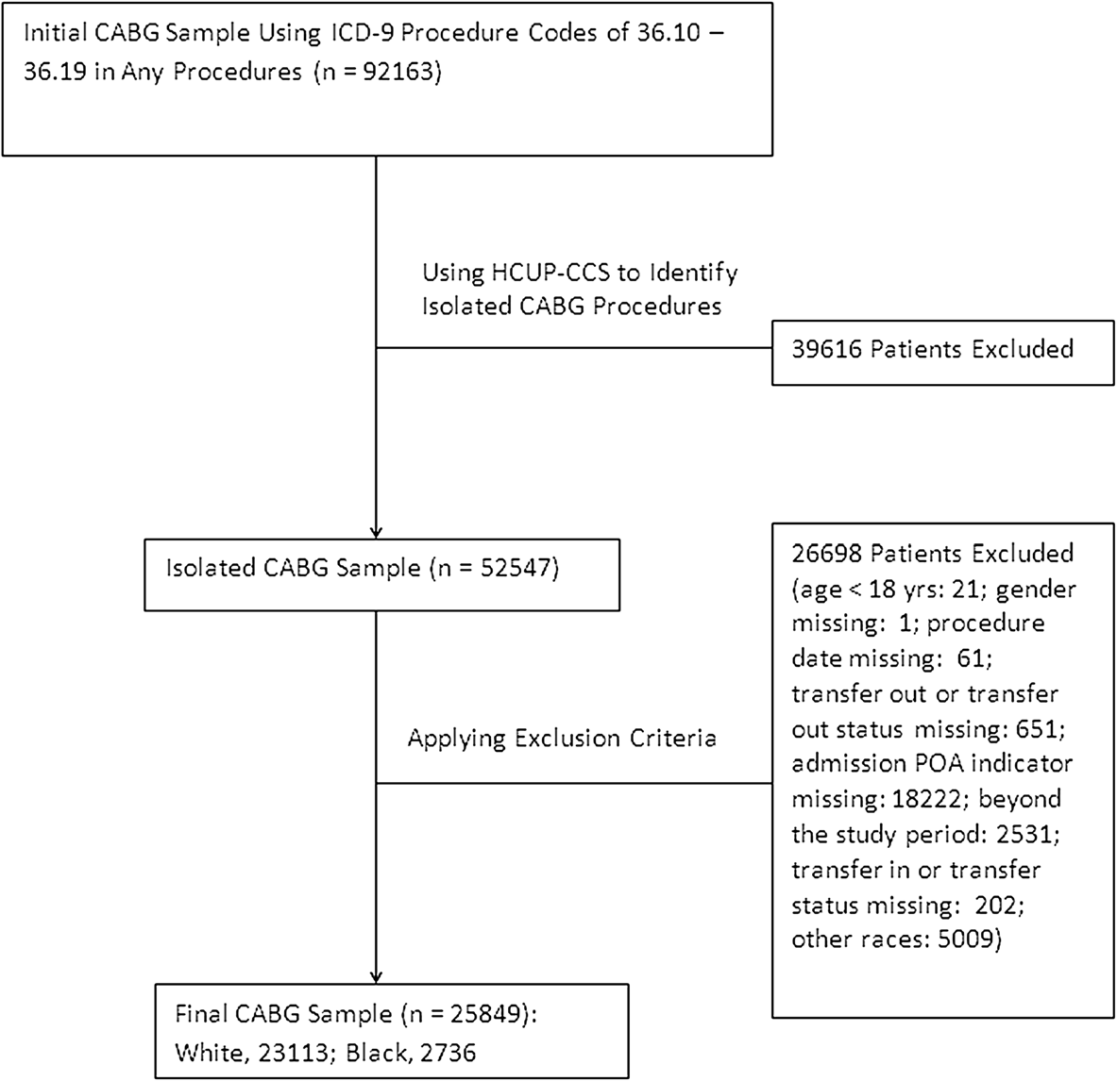
Flow chart for CABG sampling.

**Figure 2:**
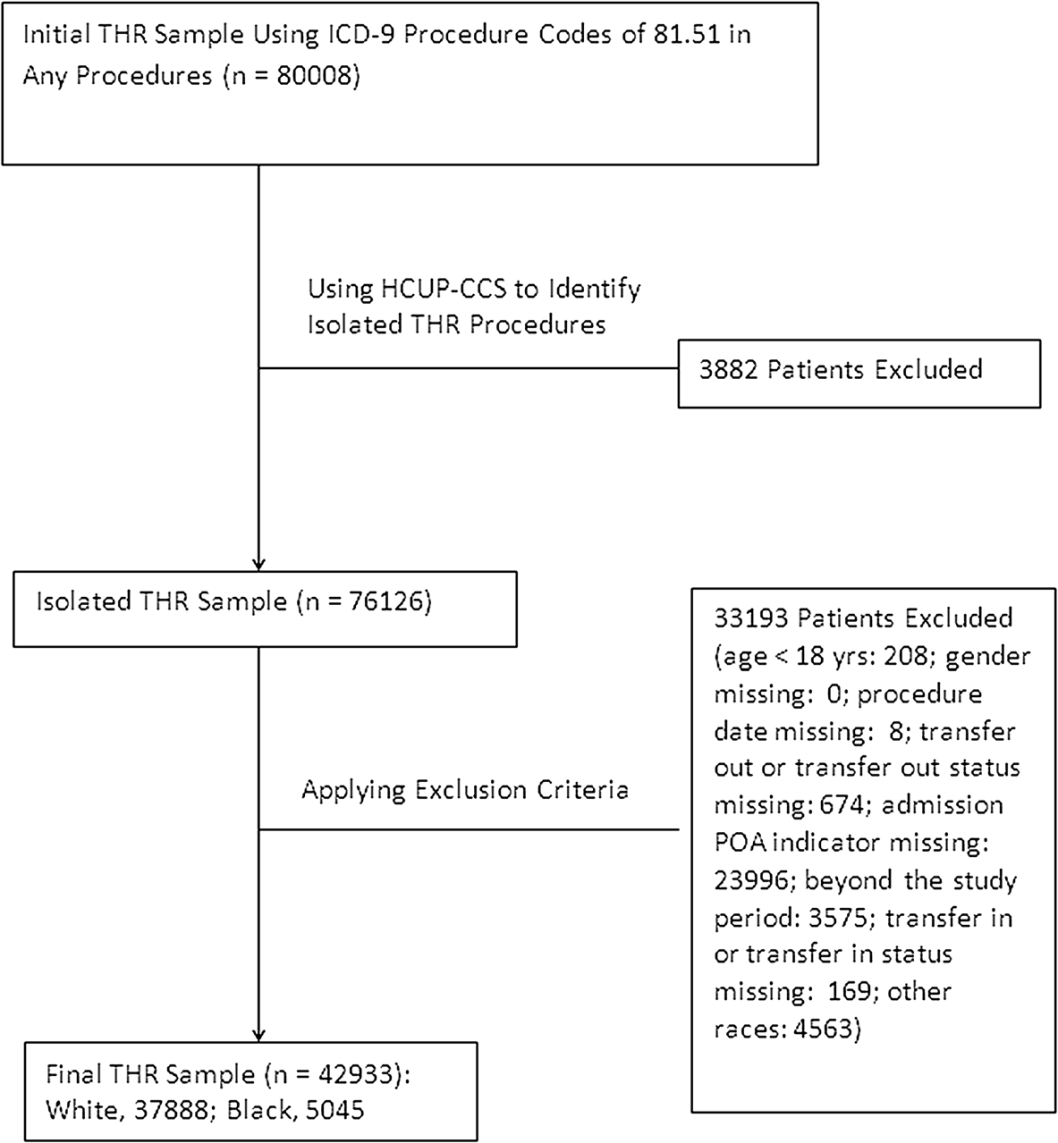
Flow chart for THR sampling.

**Figure 3:**
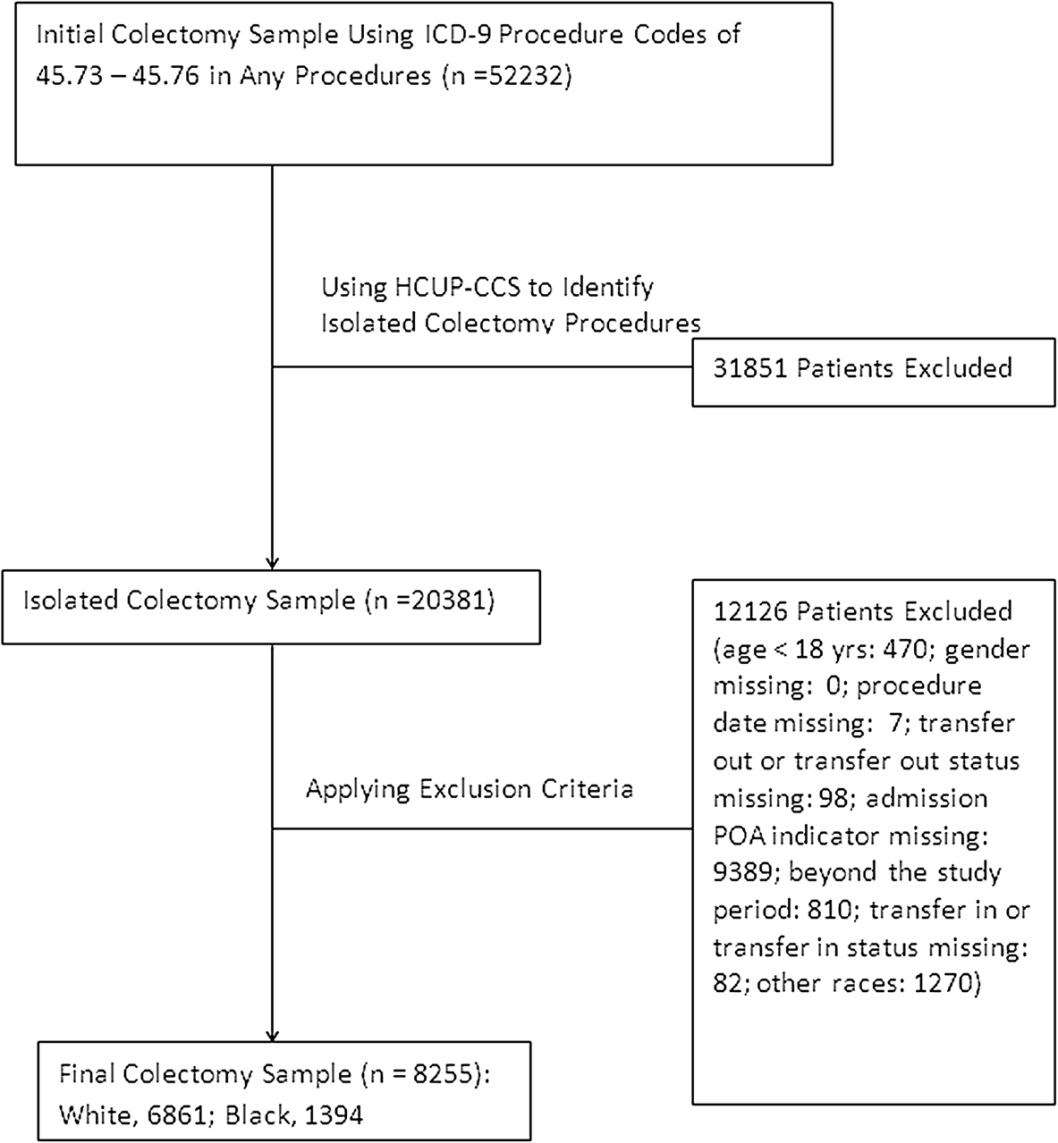
Flow chart for colectomy sampling.

Black or white patients who received one of the following three surgical procedures were included in the analysis: isolated coronary artery bypass surgery (CABG), isolated total hip replacement (THR), or isolated colectomy. We used two steps to identify the patients who underwent isolated surgery. First, we used ICD-9-CM procedure codes to identify CABG (36.10 - 36.19), THR (81.51), and colectomy (45.73-45.76). Second, we applied the Healthcare Cost and Utilization Project (HCUP) clinical classification software (CCS) for ICD-9-CM (available at: http://www.hcup-us.ahrq.gov/toolssoftware/ccs/ccs.jsp) to identify the patients who underwent primary isolated CABG, THR, or colectomy surgery during the hospitalization. These procedures were selected because they are commonly performed major surgical procedures representing cardiac surgery, orthopedics, and general surgery; and patients undergoing these procedures have relatively high transfusion rates. We excluded patients whose age was less than 18 years old and patients whose important demographic characteristics (e.g., gender) or important healthcare information (e.g., procedure date, transfer out status, transfer in status, admission POA indicator, and admission status) were missing. The final study sample comprised 25,849 isolated CABG patients, 42,933 isolated THR patients, and 8,255 isolated colectomy patients in 87 hospitals (see Figures 1, 2, 3).

### Variable definition

#### ***RBC Transfusion***

The UHC dataset contains usage information of perioperative (intraoperative and postoperative) allogeneic packed RBC transfusions.

#### ***Hospital concentration of black patients undergoing surgery***

The hospital’s concentration of black patients undergoing surgery was grouped into quartiles: quartile 1 (≤ 8%), quartile 2 (8-13%), quartile 3 (13-27%), and quartile 4 (> 27%).

### Statistical analyses

Baseline characteristics were summarized as frequency, percentage, median (inter- quartile range) as appropriate for each racial group for each surgical procedure. Comparison between racial groups (white vs. black) were made using Chi square test or Fisher exact test for categorical variables, and *t* test for continuous variables (see Table 1).

**Table 1:** Demographic characteristics of patients undergoing isolated major surgery

**Characteristics**	**CABG**		**Total hip replacement**		**Colectomy**	
	**White**	**Black**	**White**	**Black**	**White**	**Black**
	**(n = 23,113)**	**(n = 2,736)**	**(n = 37,888)**	**(n = 5,045)**	**(n = 6,861)**	**(n = 1,394)**
RBC transfused (%)	10,284 (44.5)	1,599 (58.4)	10,680 (28.2)	1,701 (33.7)	1,341 (19.6)	314 (22.5)
Age, median (IQR)	65 (58-73)	61 (55-69)	63 (55-72)	57 (49-65)	62 (50-73)	59 (48-68)
Female	5,124 (22.2)	1,135 (41.5)	20,552 (54.2)	2,738 (54.3)#	3,403 (49.6)	720 (51.7)#
Primary payer						
Private	8,802 (38.1)	761 (27.8)	17,849 (47.1)	1,772 (35.1)	3,082 (44.9)	389 (27.9)
Medicare	11,441 (49.5)	1,291 (47.2)	17,067 (45.1)	2,035 (40.3)	2,984 (43.5)	540 (38.7)
Medicaid	1,032 (4.5)	330 (12.1)	1,446 (3.8)	798 (15.8)	338 (4.9)	236 (16.9)
Self-pay	697 (3.0)	164 (6.0)	324 (0.9)	108 (2.1)	209 (3.1)	105 (7.5)
Other	1,120 (4.9)	181 (6.6)	1,163 (3.1)	324 (6.4)	241 (3.5)	122 (8.8)
Admission status						
Emergent	4,749 (20.6)	817 (29.9)	1,099 (2.9)	141 (2.8)#	1,508 (22.0)	464 (33.3)
Urgent	6,715 (29.1)	763 (27.9)	895 (2.4)	170 (3.4)	702 (10.2)	141 (10.1)#
Elective	11,615 (50.3)	1,152 (42.1)	35,818 (94.5)	4,725 (93.7)	4,602 (67.1)	763 (54.7)
Trauma	6 (0.03)	1 (0.04)	67 (0.2)	7 (0.2)#	43 (0.6)	22 (1.6)
Comorbidity						
Congestive heart failure	59 (0.3)	14 (0.5)	877 (2.3)	169 (3.4)	364 (5.3)	101 (7.3)
Valve disease	29 (0.1)	5 (0.2)#	1,475 (3.9)	96 (1.9)	326 (4.8)	45 (3.2)
Pulmonary circ disease	19 (0.1)	3 (0.1)#	300 (0.8)	76 (1.5)	104 (1.5)	31 (2.2)#
Periphery vascular disease	3,557 (15.4)	490 (17.9)	756 (2.0)	96 (1.9)#	333 (4.9)	57 (4.1)#
Hypertension	16,100 (69.7)	1,706 (62.4)	19,924 (52.6)	3,250 (64.4)	3,129 (45.6)	818 (58.7)
Paralysis	111 (0.5)	22 (0.8)	108 (0.3)	19 (0.4)#	61 (0.9)	18 (1.3)#
Other neurology disorder	715 (3.1)	84 (3.1)#	1,306 (3.5)	147 (2.9)	295 (4.3)	49 (3.5)#
Chronic pulmonary disease	4,557 (19.7)	520 (19.0)#	4,732 (12.5)	806 (16.0)	987 (14.4)	176 (12.6)#
Diabetes w/o complications	7,148 (30.9)	1,071 (39.1)	3,816 (10.1)	827 (16.4)	889 (13.0)	285 (20.4)
Diabetes with complications	1,757 (7.6)	319 (11.7)	417 (1.1)	83 (1.65)	136 (2.0)	35 (2.5)#
Hypothyroid	2,316 (10.0)	160 (5.9)	5,082 (13.4)	288 (5.7)	703 (10.3)	64 (4.6)
Renal failure	3,129 (13.5)	711 (26.0)	1,321 (3.5)	357 (7.1)	395 (5.8)	123 (8.8)
Liver disease	411 (1.8)	58 (2.1)#	535 (1.4)	127 (2.5)	170 (2.5)	26 (1.9)#
Peptic ulcer	9 (0.04)	0 (0.0)#	7 (0.02)	0 (0.0)#	3 (0.04)	1 (0.07)#
AIDS	27 (0.12)	15 (0.6)	88 (0.2)	69 (1.4)	8 (0.1)	7 (0.5)
Lymphoma	122 (0.5)	11 (0.4)#	240 (0.6)	34 (0.7)#	47 (0.7)	7 (0.5)#
Metastatic cancer	50 (0.2)	3 (0.1)#	225 (0.6)	21 (0.4)#	962 (14.0)	208 (14.9)#
Tumor w/o metastasis	328 (1.4)	44 (1.6)#	263 (0.7)	23 (0.5)	205 (3.0)	43 (3.1)#
Rheumatoid arthritis	478 (2.1)	66 (2.4)#	1,460 (3.9)	335 (6.6)	179 (2.6)	31 (2.2)#
Coagulopathy*	121 (0.5)	12 (0.4)#	183 (0.5)	14 (0.3)	32 (0.5)	4 (0.3)#
Obese	4,564 (19.8)	566 (20.7)#	4,955 (13.1)	864 (17.1)	696 (10.1)	178 (12.8)
Weight loss	204 (0.9)	34 (1.2)#	110 (0.3)	26 (0.5)	317 (4.6)	91 (6.5)
Fluid electrolyte disorder	1,112 (4.8)	195 (7.1)	718 (1.9)	108 (2.1)#	467 (6.8)	149 (10.7)
Blood loss anemia	48 (0.2)	14 (0.5)	62 (0.2)	9 (0.2)#	93 (1.4)	47 (3.4)
Deficiency anemia	1,880 (8.1)	476 (17.4)	1,913 (5.1)	423 (8.4)	934 (13.6)	262 (18.8)
Alcohol abuse	804 (3.5)	135 (4.9)	693 (1.8)	137 (2.7)	167 (2.4)	66 (4.7)
Drug abuse	296 (1.3)	161 (5.9)	334 (0.9)	170 (3.4)	85 (1.2)	58 (4.2)
Psychoses	374 (1.6)	49 (1.8)#	657 (1.7)	119 (2.4)	154 (2.2)	49 (3.5)
Depression	1,906 (8.3)	149 (5.5)	4,660 (12.3)	394 (7.8)	628 (9.2)	68 (4.9)

Unless otherwise indicated, results are presented as number of patients and percentages.

*Abbreviations*: *IQR* inter-quartile range, *AIDS* acquired immune deficiency syndrome. All p values < 0.05 except marked with #.

*This refers to the newly defined coagulopathy used in the analysis, which recoded ICD-9 codes of 286.6, 286.7, 286.9, 287.4, and 287.5 as 0 for this comorbidity variable. The reason for the recoding is that the coagulopathy codes could be complications related to blood transfusion.

The outcome variable was whether a patient received any perioperative RBC transfusion. A patient-level binary variable was coded as 1 if the patient received RBC transfusion perioperatively and as 0 otherwise. The primary independent variable of interest was race (white vs. black). The unit of analysis in the study was the patient.

To examine racial disparities in blood transfusion, a series of multivariable logistic regression models for each surgical procedure were fit to the patient-level data in a sequential manner. The baseline model (model 1) examined the association between race and transfusion, controlling for patient demographics (age and gender), admission status, APR-DRG severity of illness, POA status, and AHRQ comorbidities (including anemia). To determine whether black-white differences in transfusion persisted within payer groups, we interacted race and payer status (model 2).

In order to examine whether the source of black-white differences in blood transfusion was due to between-hospital or within-hospital effects, we estimated two additional models. In the first model, we added dummy variables representing the hospital quartile of black patient concentration to the baseline model (model 3). In the second model, we added hospital fixed effects to the baseline model (model 4). Robust variance estimators were used because patient outcomes within the same hospital may be correlated [41]. Data management, analyses, and regression diagnostics were performed using Stata MP version 11.0 (StataCorp, College Station, TX). All statistical tests were 2-sided and P < 0.05 was considered significant.

## Results

In comparison with whites, black patients were younger, less well-insured, had more co-morbidities (Table 1). Black patients were more likely to receive perioperative RBC transfusions for two of the three procedures examined. For isolated CABG, 58.4% of black patients received one or more units of red blood cells, compared to 44.5% for white patients (CABG: OR = 1.75, 95% CI: [1.42, 2.17], p < 0.001). For isolated THR, 33.7% of black patients received RBC transfusion, compared to 28.2% for white patients (THR: OR = 1.30, 95% CI: [1.11, 1.51], p = 0.001). For isolated colectomy, 22.5% of black patients received one or more units of RBCs, compared to 19.6% for white patients (Colectomy: OR = 1.20, 95% CI: [0.99, 1.44], p = 0.053) (Table 2).

**Table 2:** Risk-adjusted RBC transfusion in major surgery as a function of race

**Major surgery**	**Unadjusted**		**Baseline**^**a**^		**Within Hospitals**^**b**^	
	**OR (95% CI)**	**P value**	**AOR (95% CI)**	**P value**	**AOR (95% CI)**	**P value**
CABG						
Whites	(Reference)		(Reference)		(Reference)	
Blacks	1.75 (1.42-2.17)	<0.001	1.41 (1.13-1.76)	0.002	1.42 (1.30-1.56)	<0.001
THR						
Whites	(Reference)		(Reference)		(Reference)	
Blacks	1.30 (1.11-1.51)	0.001	1.39 (1.20-1.62)	<0.001	1.43 (1.29-1.58)	<0.001
Colectomy						
Whites	(Reference)		(Reference)		(Reference)	
Blacks	1.20 (0.997-1.44)	0.053	1.08 (0.90-1.30)	0.40	1.01 (0.85-1.19)	0.92

OR: odds ratio, AOR: adjusted odds ratio, CI: confidence interval, CABG: coronary artery bypass surgery, THR: total hip replacement.

^a^Indicates patient level risk adjustment model (age, gender, admission status, APR-DRG severity of illness, and AHRQ comorbidities).

^b^Indicates within-hospital risk adjustment model using hospital fixed effects model (all patient risk factors above plus hospital fixed effects).

After adjusting for patient level covariates, black patients undergoing CABG surgery had a 41% higher odds of receiving perioperative RBC transfusion (AOR = 1.41, 95% CI: [1.13, 1.76], p = 0.002), and blacks undergoing THR had a 39% higher odds of transfusion (AOR = 1.39, 95% CI: [1.20, 1.62], p < 0.001) compared to white patients. We did not detect significant racial difference in transfusion for isolated colectomy (AOR = 1.08, 95% CI: [0.90, 1.30], p = 0.40) (Table 2).

**Figure 4:**
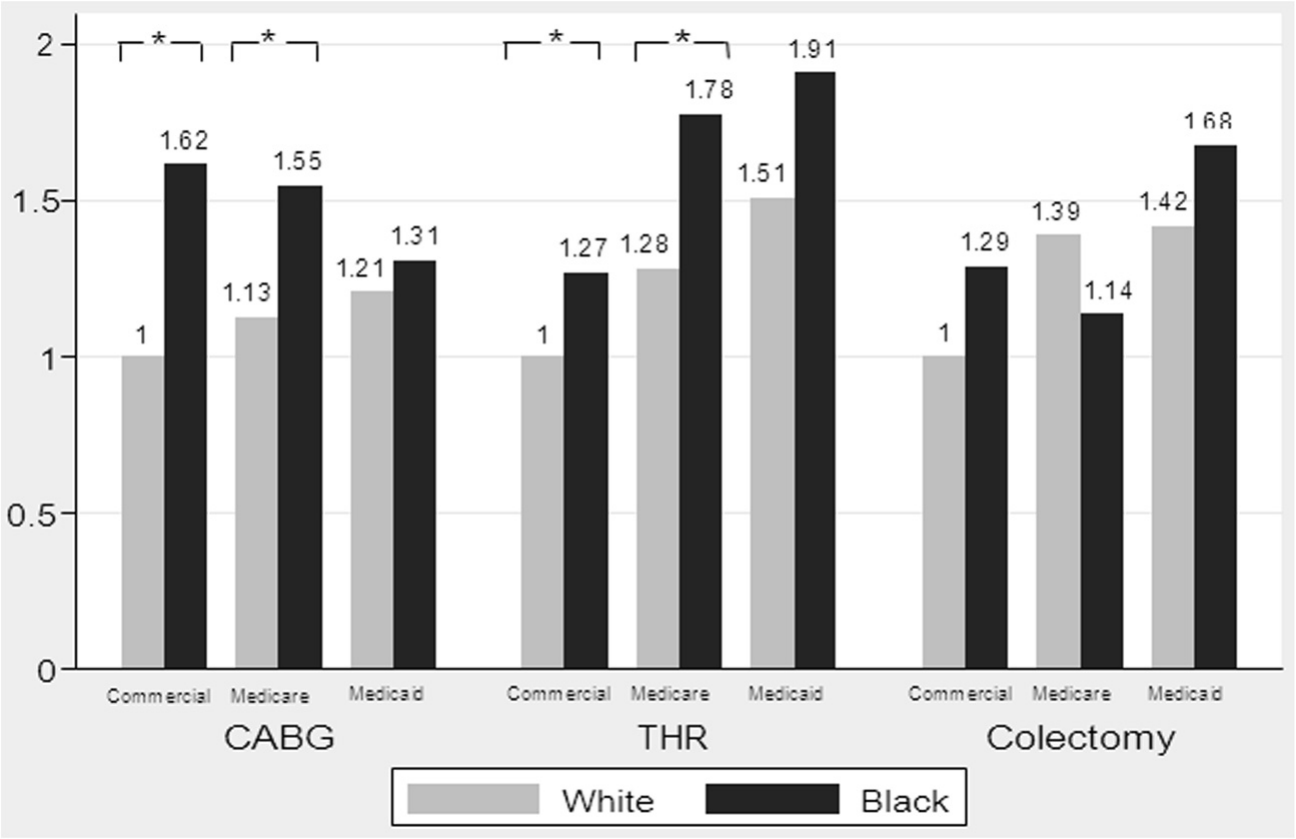
**Impact of patient insurance on racial disparities in the use of blood transfusion in major surgery.** AOR: Adjusted odds ratio; *p<0.05 for pairwise comparison with identical insurance coverage. This figure is based on the risk adjusted models controlling for patient-level factors (age, gender, admission status, APR-DRG severity of illness, and AHRQ comorbidities) and full interaction terms between race (black vs. white) and patient insurance.

After also adjusting for insurance status, black-white differences in transfusion still existed for patients with identical insurance coverage. Compared to white patients with private insurance, black patients with private insurance had 62% and 27% increased odds of being transfused for isolated CABG (AOR = 1.62, 95% CI: [1.33, 1.97], p < 0.001) and isolated THR (AOR = 1.27, 95% CI: [1.06, 1.52], p = 0.01), respectively. Compared to white patients with Medicare, black patients with Medicare had 37% and 39% higher odds of receiving transfusion for isolated CABG (AOR = 1.37, 95% CI: [1.04, 1.79], p = 0.02) and isolated THR (AOR = 1.39, 95% CI: [1.18, 1.62], p < 0.001), respectively (Figure 4). Black-white differences in blood use were very similar before and after controlling for insurance status (results not shown).

We did not find evidence of an association between blood use and the hospital concentration of black patients for patients undergoing THR or CABG surgeries (Table 3). Finally, black-white differences in the use of perioperative RBC transfusion remained almost unchanged after we included hospitals fixed effects (Table 2). These results suggest that black-white differences in blood use were more likely to be caused by “within hospital variation” rather than “across hospital variation” [2,42]. Each of the multivariable regression models exhibited good discrimination: the Cstatistics were all greater than 0.72. The Hosmer-Lemeshow statistic ranged between 4.16 and 44.8, consistent with acceptable model calibration given the well-known sensitivity of the Hosmer-Lemeshow statistic to sample size [43].

**Table 3:** Association between RBC transfusion and hospital concentration of black patients

**Hospital concentration of black patients**	**CABG**		**THR**		**Colectomy**	
	**AOR (95% CI)**	**P value**	**AOR (95% CI)**	**P value**	**AOR (95% CI)**	**P value**
Q1 (Low: ≤8%)	(Reference)		(Reference)		(Reference)	
Q2 (Medium: 8-13%)	0.85 (0.56-1.30)	0.46	1.07 (0.66-1.74)	0.79	0.89 (0.61-1.29)	0.54
Q3 (Medium high: 13-27%)	1.06 (0.68-1.66)	0.79	1.06 (0.63-1.80)	0.82	1.27 (0.91-1.76)	0.15
Q4 (High: >27%)	1.29 (0.73-2.28)	0.38	1.24 (0.74-2.10)	0.41	1.48 (1.07-2.04)	0.02

AOR: adjusted odds ratio, CI: confidence interval, CABG: coronary artery bypass surgery, THR: total hip replacement.

## Discussion

Using a contemporary nationwide database and controlling for patient-level factors, we demonstrated that black patients undergoing CABG and total hip replacement in academic medical centers were 40% more likely to receive a blood transfusion compared to white patients. This black-white gap in transfusion practice persisted after adjusting for patient insurance status. Our analyses show that racial disparities in blood transfusion in major surgery exist and that such disparities reflect differences in transfusion practices for black and white patients within the same hospital. Greater use of blood transfusions in black patients may reflect lower surgical technical quality leading to greater blood loss in black compared to white patients [44].

### Almost a decade ago, the Institute of Medicine (IOM) report *unequal treatment*

*Confronting Racial and Ethnic Disparities in Healthcare* summarized the striking finding that blacks tended to receive lower quality healthcare across a wide range of clinical services. For example, blacks were more likely to receive lower-extremity amputations [45]. This influential report also concluded that the racial differences may stem from patient characteristics (e.g., minority patients may be more likely to delay seeking healthcare), clinical encounters (e.g., medical uncertainty can “open the door” for physicians’ stereotypes and biases to affect their clinical judgment of minority patients), and health systems (e.g., fragmented healthcare delivery systems present cultural and linguistic barriers for minority patients). In response, numerous high-profile initiatives at both national and local levels were designed and implemented to mitigate or eliminate racial and ethnic disparities in healthcare. However, the *2010 National Healthcare Disparities Report* demonstrated that racial disparities in healthcare have persisted despite these efforts [15].

The stubborn persistence of racial disparities over time may, in part, reflect a lack of understanding of the underlying causes of racial disparities in healthcare. More recently, a new explanation for racial disparities in health care has drawn great attention because it leads to different policy implications to overcome disparities. Supported by empirical studies, this explanation proposes that racial and ethnic disparities are determined by both discrimination (i.e., “within provider variation”, “who you are”) and segregation (i.e., “across provider variation”, “where you live”) [2,7,12,42,46]. The former highlights the importance of race in the clinical encounter, whereas the latter relates more to geographic variations in the quality of care patterns for all patients. Using this framework, new studies have found that some types of racial disparities in healthcare were largely caused by discrimination [47,48] while others were mainly attributable to segregation [21,49]. To reduce the former type of racial disparities, efforts to improve patient-physician communication and to enhance “patient-centered” care during the clinical encounter are recommended. These efforts include physician cultural competency training, expansions in the numbers of minority physicians in the hospital, hospital’s adoption of patient-centered information technology, and hospital’s efforts to improve effective communication and to promote “communities of care” [45,50-53]. To reduce latter type of racial disparities, interventions to improve performance of particular hospitals which served disproportionately high concentrations of minority patients but provided suboptimal quality of care are needed. These interventions could involve system-level quality initiatives such as pay for performance to incentivize quality improvement in low-quality black-serving hospitals [49,54].

Our findings of racial disparities in transfusion in isolated CABG and THR surgeries not only fill the knowledge gap of contemporary transfusion practices but also suggest the main cause of such disparities - discrimination (“within hospital variation”) during the clinical encounter. This may reflect, in part, complexity of the medical decision making process underlying transfusion decisions. The decision to transfuse a patient can be complex (whether, what, and how much to transfuse) and can involve more than one physician (surgeon, anesthesiologist, critical care medicine physician). Some of the variation in transfusion practices may be due to variation in surgical blood loss, and may account for some of the racial variation in the use of blood transfusion. Surgical procedure time, surgeon technical skill, case complexity, and anesthetic management can contribute to the variation in operative blood loss in complex ways. For example, Silber et al reported that black race was associated with increased anesthesia time and significantly longer procedure time in noncardiac surgery [55]. Our results also indicate that patient insurance coverage and racial segregation (“across hospital variation”) were not major contributors to the detected racial disparities in blood transfusion. Therefore, improving insurance coverage and focusing on black-serving hospitals only might have very limited power in reducing racial disparities in blood transfusion.

Our findings have important policy implications. First, as blood transfusion is gaining recognition as an important domain of quality of care, our study suggests that efforts to monitor and reduce racial disparities in transfusion practices are necessary and important. Since blood transfusion is widely employed in surgery, improving quality of care in transfusion therapy with a particular focus on minorities may lead to improved perioperative outcomes. Second, given that these racial gaps are largely due to “within hospital variation”, interventions with the goal of improving patient-physician communication and enhancing patient-centered health care are more likely to be effective and successful in narrowing these disparities. Fueled by the Patient Protection and Affordable Care Act’s emphasis on patient preferences and medical decision making, there is growing enthusiasm in promoting patient-centered care and addressing racial and ethnic disparities. Third, our findings provide further empirical evidence of black-white disparities in health care. Historically, minorities tend to receive fewer interventions associated with improved health outcomes (e.g. PCI for acute myocardial infarction) [4,5]. However, in some cases, black patients are more likely to undergo interventions suggestive of less than optimal management of chronic diseases (e.g. it was also reported that black patients had significantly higher rates of lower-extremity amputations) [14,15]. Since blood transfusion is frequently associated with higher risk of mortality and complications in surgical patients, our findings that black patients undergoing surgery are more likely to receive blood transfusion suggests there is a need to examine the clinical appropriateness of transfusion therapy in minority patients [56,57].

Our study has significant limitations. Most importantly, preoperative hematocrit values were not available in the UHC database. We were able to control only for the presence but not for the severity of anemia. Thus it is likely that some of the black-white differences in blood transfusions may be due to unmeasured variation in patient preoperative hematocrits. Second, it is possible that a small portion of blacks could be misclassified as whites and vice versa [58]. Thus, our results could be biased towards the null and may underestimate the actual racial disparities in blood transfusion. Third, the present study is based on an administrative database. Therefore, it shares drawbacks of using administrative datasets in evaluating quality such as inability to specify clinical definitions for risk factors (e.g., forced reliance on ICD-9 codes), and limited ability to distinguish between complications of care and pre-existing conditions. Fourth, we cannot rule out the possibility of residual confounding because of other unmeasured risk factors which could account for some of the black-white differences in blood transfusion. Fifth, the blood usage information in the UHC database has not been audited. Finally, because only academic hospitals are included in the UHC database, our findings are not necessarily generalizable to non-academic centers.

## Conclusions

In summary, black patients are more likely than white patients to receive blood transfusion for CABG and THR surgery, even after controlling for patient factors and insurance status. Furthermore, these racial disparities are most likely due to differences in transfusion practices between black and white patients within the same hospitals, rather than due to black patients undergoing surgery at predominantly black-serving hospitals. Since a lack of awareness of racial disparities in healthcare remains a significant barrier to eliminating unequal treatment [45,59], reporting racial disparities in contemporary transfusion practices may help reduce potentially unnecessary blood transfusions in minority patients.

## Competing interests

The authors declared that they have no competing interests.

## Authors’ contributions

FQ, ME, SL, and LG conceived the research idea, FQ, ME, SL, SH, CD, RP, RW, and LG participated in its design, SH and LG acquired the data, FQ and LG performed the statistical analysis and drafted the manuscript, ME, SL, SH, CD, RP, RW gave critical comments and helped revising the manuscript. All authors read and approved the final manuscript.

## Pre-publication history

The pre-publication history for this paper can be accessed here:

http://www.biomedcentral.com/1472-6963/14/121/prepub
